# Supplementary material for: Hardening of Respiratory Syncytial Virus Inclusion Bodies by Cyclopamine Proceeds through Perturbation of the Interactions of the M2-1 Protein with RNA and the P Protein
Source: Int J Mol Sci. 2023 Sep 8;24(18):13862. doi: 10.3390/ijms241813862 (PMC10531356; doi:10.3390/ijms241813862)
Supplement: Supplementary file 1 [file ijms-24-13862-s001.zip › ijms-2494477-supplementary.pdf]

Supplemental Materials

Hardening of Respiratory Syncytial Virus Inclusion Bodies by Cyclopamine Proceeds through Perturbation of the Interactions of the M2-1 Protein with RNA and the P Protein

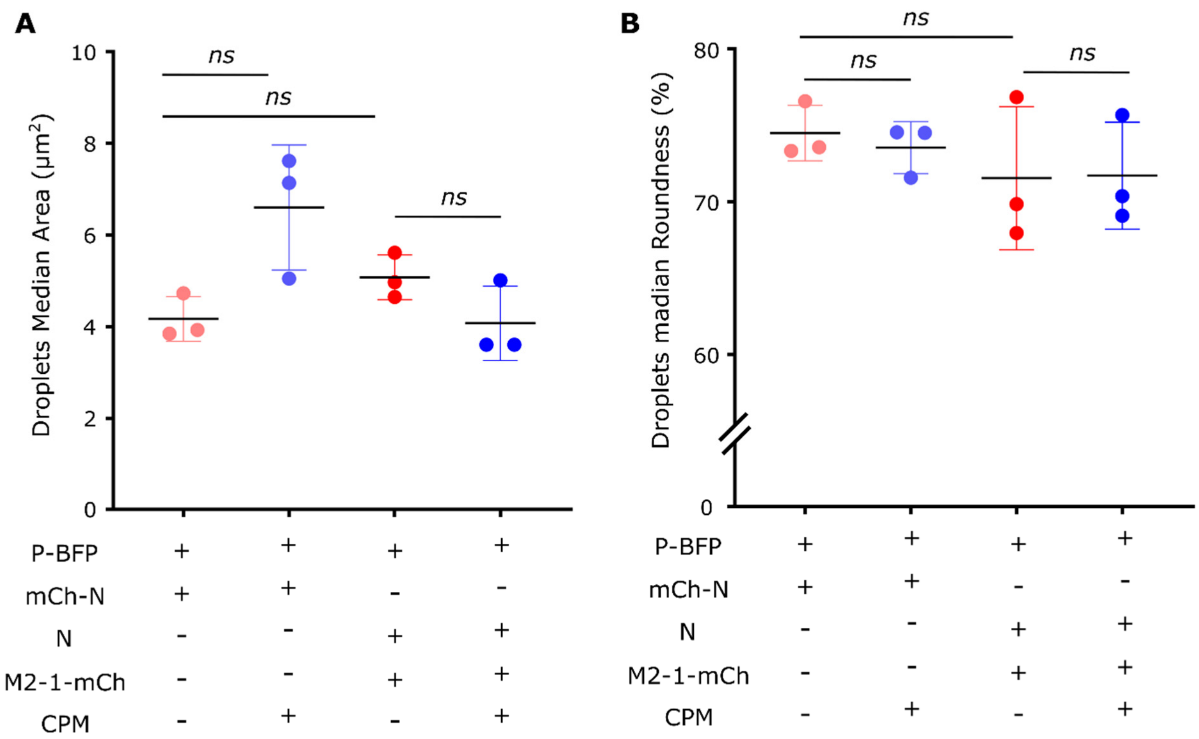

Figure S1. Droplets size is not altered by M2-1 or CPM addition. Size and roundness of condensates were quantified on the Icy software using the Analysis Particles function and a size threshold at >3 µm². Imaging was performed under a Leica SP8 microscope using the 63x oil-immersion objective. Artefactual values from aggregates and pairs of condensates fusing together were filtered-out using a 0.9 sphericity cut-off. These parameters were measured on 500 to 1,500 condensates per experiment. Median area (A) and roundness (B) from three independent experiment are plotted, central bars indicate mean ± SD. Statistical analysis were done using Kolmogorov-Smirnov test; *ns*: not significant.

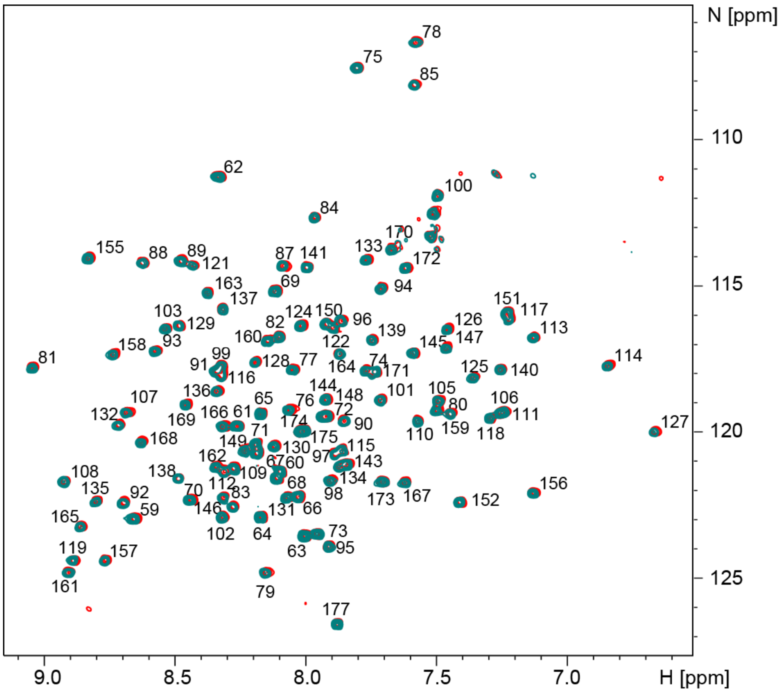

Figure S2: CPM does not bind to M2-1core with high affinity. ^{1}H-^{15}N BTROSY spectra of 100 µM ^{15}N-labeled M2-1core domain without (red contours) and with one molar equivalent of CPM (teal contours) are superimposed. Measurements were done at 700 MHz ^{1}H frequency and at a temperature of 298K. Addition of CPM does not induce significant chemical shift or intensity perturbations, indicating that CPM does not bind to M2-1core. Amide signals are annotated with the corresponding residue number.

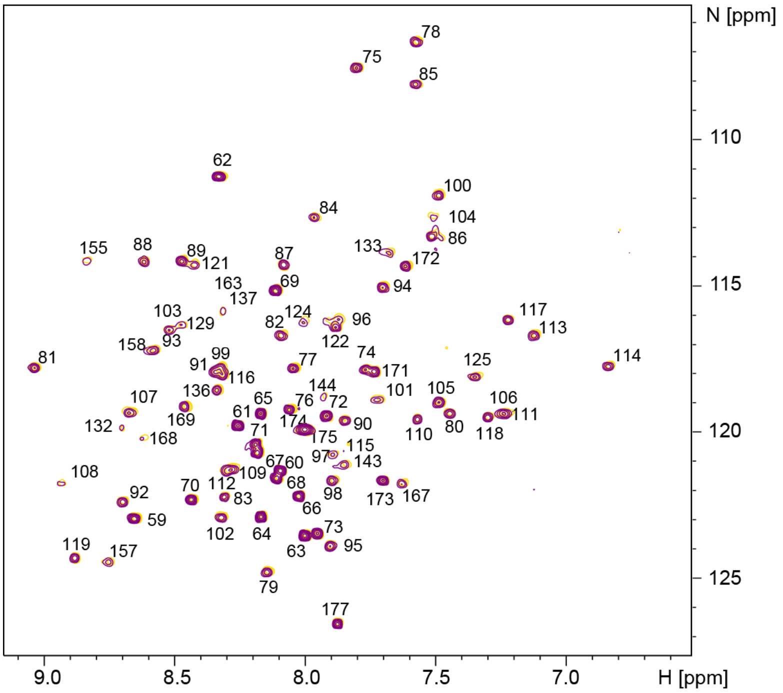

Figure S3: CPM does not perturb the complex between ^{15}N-labeled M2-1core domain and the FITC-P$_{95-112}$ peptide. ^{1}H-^{15}N BTROSY spectra of ^{15}N-labeled wild-type M2-1core domain (100 µM) in complex with FITC-P$_{95-112}$ peptide in a 0.25:1 ratio before (yellow) and after (purple) addition of 1 molar equivalent of CPM (100 µM final concentration). Measurements were done at 700 MHz ^{1}H frequency and at a temperature of 298K.

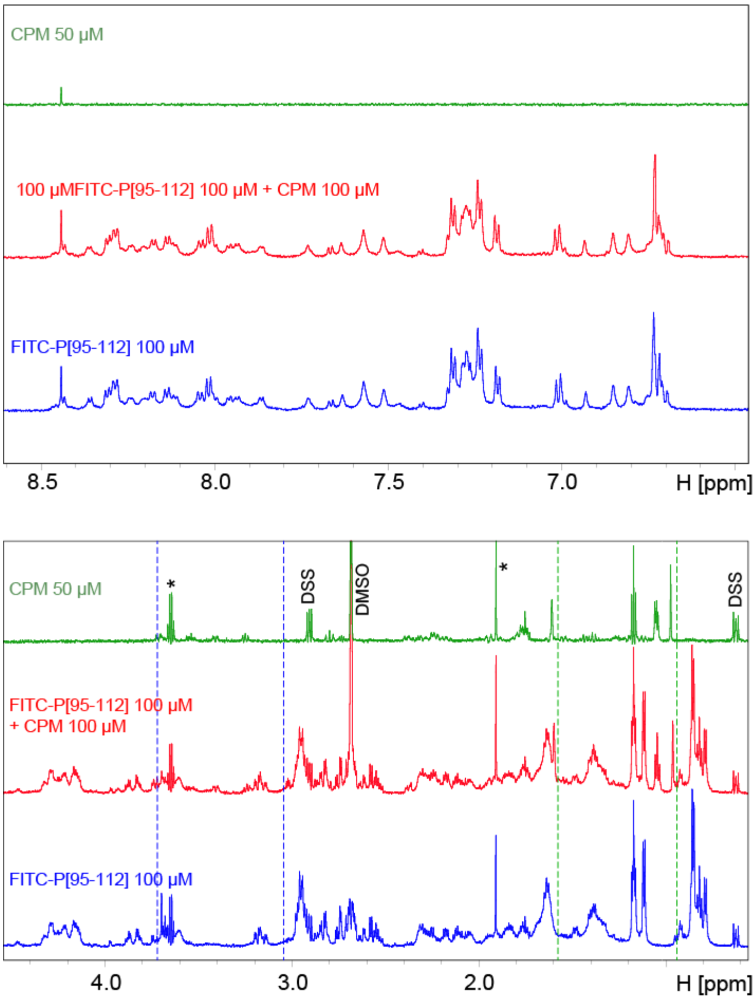

Figure S4: Analysis of the interaction between FITC-P95-112 and CPM by NMR. 1D ^{1}H NMR spectra were acquired with water suppression by excitation sculpting at 700 MHz ^{1}H frequency and at a temperature of 298K for FITC-P95-112 alone (100 µM), CPM alone (50 µM) and an equimolar mixture of both (100 µM both), under the same buffer conditions (phosphate saline at pH 6.8). Samples with CPM contained 0.8 % DMSO-d6. 50 µM DSS was used as a chemical shift reference. The DSS signal at 0 ppm is not shown, but the DSS signals around 0.65 and 2.9 ppm are indicated. Impurities contained in the buffer are indicated by a star. Vertical lines indicate displacement of CPM (green) FITC-P95-112 (blue) signals.

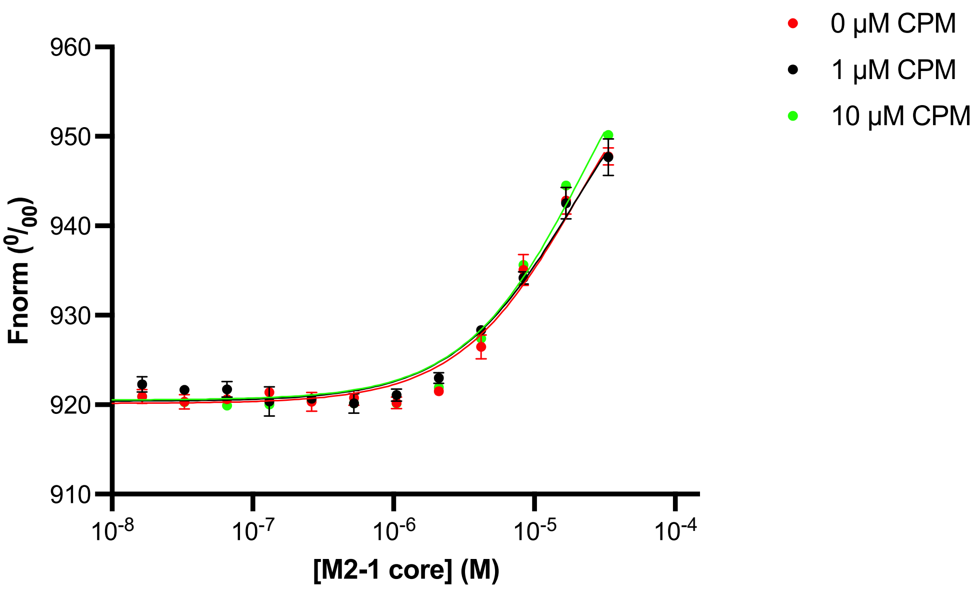

Figure S5: CPM does not affect the affinity of the complex between M2-1 core domain and P90-112 peptide. Microscale Thermophoresis analysis of the titration of 100 nM FITC-P90-112 peptide with an increasing amount of

M2-1core domain, either in absence of CPM (red dots) or in presence of 1 µM (black dots) or 10 µM (green dots) CPM. Data are presented as mean ± SD of two independent experiments, except for the condition 10 µM which was tested one time. They were analysed using the temperature-jump phase and the standard fitting mode derived from law of mass action.

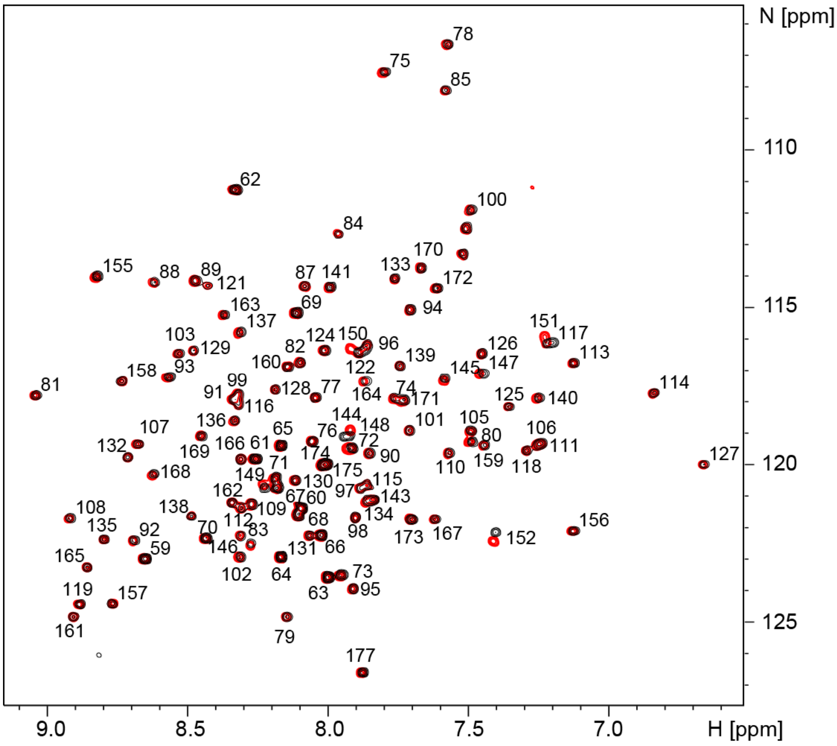

Figure S6: Impact of the R151K mutation on the structure of M2-1. ^{1}H-^{15}N BTROSY spectra of ^{15}N-labeled WT (red contours) and R151K (black contours) M2-1core domain are superimposed. Measurements were done at 700 MHz ^{1}H frequency and at a temperature of 298K. The R151K mutation induces only small chemical shift perturbations around the mutated residue. Amide signals are annotated with the corresponding residue number.

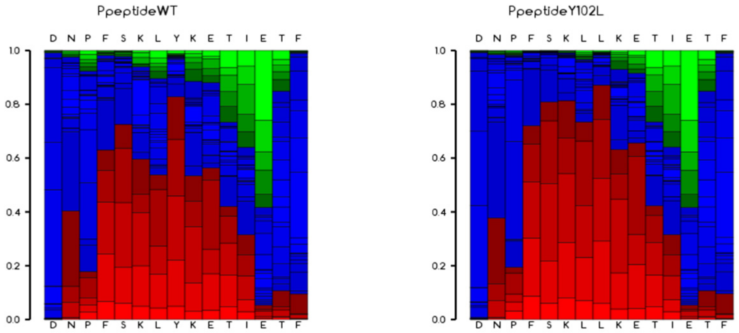

Figure S7: Impact of the Y102L mutation on the conformation of the M2-1 binding motif in RSV P. Secondary structure propensities were predicted using PEP-FOLD 3.5 for peptides containing residues 95-109 of P. The probabilities of helical (red), coil (blue) and extended (green) secondary structure are shown in bar diagrams for each residue in wild-type and the Y102L mutant. Both peptides are predicted with high α-helical propensity.
